# Peripheral enthesitis assessed by whole-body MRI in axial spondyloarthritis: Distribution and diagnostic value

**DOI:** 10.3389/fimmu.2022.976800

**Published:** 2022-08-23

**Authors:** Zikang Guo, Boya Li, Yimeng Zhang, Chunyu Kong, Yang Liu, Jin Qu, Ying Zhan, Zhiwei Shen, Xinwei Lei

**Affiliations:** ^1^ First Central Clinical College, Tianjin Medical University, Tianjin, China; ^2^ Department of Rheumatology, Tianjin First Central Hospital, Tianjin, China; ^3^ Department of Radiology, Tianjin First Central Hospital, Tianjin Institute of Imaging Medicine, Tianjin, China; ^4^ Clinical Science, Philips Healthcare, Beijing, China

**Keywords:** axial spondyloarthritis, whole-body MRI, enthesitis, diagnosis, ROC (receiver operating characteristic curve)

## Abstract

**Objective:**

To determine the distribution and diagnostic value of peripheral enthesitis detected by whole-body MRI (WBMRI) in axial spondyloarthritis (axSpA) diagnosis, and to determine the value of the peripheral enthesitis score in axSpA assessment.

**Methods:**

Sixty axSpA patients [mean age of 33.2 (24.8–40.6) years] and 50 controls with chronic low back pain (LBP) [mean age of 34.7 (28.3–41.1) years] were enrolled. The gold standard was physician’s comprehensive diagnosis based on current classification criteria and physical examination. All subjects underwent WBMRI, and 47 peripheral entheses were assessed for each patient with scores of 0–188.

**Results:**

WBMRI identified 155 enthesitis sites in 78.3% (n = 47) patients with axSpA. Meanwhile, 23 enthesitis sites were identified in 32% (n = 16) controls. The pelvis had the maximum number of enthesitis sites (52, 33.5%) in axSpA patients. Pelvic and anterior chest wall enthesitis had the highest sensitivity (51.67%) and specificity (100%) in axSpA diagnosis, respectively. There were different manifestations of enthesitis subtypes between axSpA patients and the control group. Osteitis was more present than soft-tissue inflammation in axSpA patients. The AUC for the number of enthesitis sites was 0.819 (95% CI 0.739–0.899), and that for the enthesitis score was 0.833 (95% CI 0.755–0.910), indicating statistically significant differences (P = 0.025). Based on the Youden index and clinical need, three enthesitis sites (sensitivity of 53.33, specificity of 98, and Youden index of 0.51) and enthesitis score (sensitivity of 58.33, specificity of 98, and Youden index of 0.56) may have the greatest value for axSpA diagnosis.

**Conclusion:**

The distribution of peripheral enthesitis can be adequately assessed by whole-body MRI, which could help diagnose axial spondyloarthritis. The enthesitis score may provide a more accurate assessment and diagnostic tool in axSpA compared with enthesitis site counting.

## Introduction

Axial spondyloarthritis (axSpA) is an inflammatory disorder of the axial skeleton associated with significant pain and high disability rate. Delayed diagnosis is associated with poorer outcomes, including functional impairment and declined quality of life. A meta-analysis reported a pooled mean diagnosis delay of 6.7 years, with high levels of heterogeneity (1). The absence of extra-articular manifestations is considered a cause of prolonged delay (2).

Correct and timely diagnosis could help start early treatment and improve patient outcomes. The classification criteria developed for axSpA in 2009, termed the Assessment of Spondyloarthritis international Society (ASAS) classification criteria (3), are the main basis for helping doctors to make a judgment at this stage. However, sacroiliitis appearance on MRI is sometimes atypical, and a Dutch study reported inflammatory lesions at the sacroiliac joints in healthy individuals, runners, and women with postpartum back pain. Interestingly, a substantial proportion of this population had MRI positivity for sacroiliitis according to the ASAS definition (4). Although spinal lesions (particularly vertebral corner inflammation or structural changes) in axSpA patients have sufficient specificity in distinguishing them from non-axSpA patients, spinal lesions do not contribute much to the classification of axSpA cases. The reason is that positivity for the spine is rare in axSpA patients without sacroiliitis on MRI and X-ray images (5, 6).

Enthesis represents the insertion of tendons and ligaments into the bone surface (7). Enthesitis is a distinctive pathological feature of spondyloarthritis and may affect synovial joints, cartilaginous joints, syndesmoses, and extra-articular entheses (8). The ASAS classification criteria only included heel enthesitis, and the diagnostic values at other peripheral locations need further evaluation. Detection of enthesitis at various sites throughout the whole body may facilitate the diagnosis of spondyloarthritis. Whole-body MRI (WBMRI) allows the visualization of the entire body in once examination. Most WBMRI studies focused on the detection of enthesitis at various sites throughout the body and on follow-up after treatment. However, relatively few studies have focused on the diagnostic value of peripheral enthesitis (9, 10).

The aim of this study was to determine the distribution and diagnostic value of peripheral enthesitis detected by WBMRI in axSpA diagnosis. In addition, we aimed to determine the value of the peripheral enthesitis score in the assessment of axial spondyloarthritis.

## Materials and methods

### Subjects

This study was approved by the Ethics Committee of Tianjin First Central Hospital (No. 2021N156KY). From June 2021 to December 2021, 120 patients with chronic low back pain (LBP) who visited the Rheumatology clinic of Tianjin First Central Hospital were analyzed. The patients underwent complete imaging (MRI and/or CT of the sacroiliac joint) and serological examinations. Written informed consent was obtained from all participants before any procedure.

Inclusion criteria were as follows. Patients diagnosed with axial spondyloarthritis by the reference standard were included in the positive group. Other patients not diagnosed with axSpA were included in the control group. The reference standard was physician’s final axSpA diagnosis based on the clinical, laboratory, and imaging information of patients who fulfilled the classification criteria [the Assessment of Spondyloarthritis International Society (ASAS) classification criteria for axSpA or the Ankylosing spondylitis (AS) criteria prescribed by the New York criteria of 1984 (11)].

Exclusion criteria were (1) >45 years of age; (2) serious primary diseases such as cardiocerebrovascular, digestive, hematopoietic system, liver, and renal diseases; (3) being athletes or practitioners in the fitness industry; (4) a history of fracture, joint surgery, or joint replacement; (5) glucocorticoid use in the last 3 months; and (6) contraindications for MRI (e.g., pacemaker use).

Totally 10 patients were excluded for fracture of the proximal tibia (one patient), engaging in sports and fitness (three patients), glucocorticoid use (two patients), and inability to complete scans (four patients). Eventually, 60 patients were diagnosed with axSpA (26 AS and 34 nr-axSpA cases). Non-radiographic axial SpA (nr-axSpA) was included in the concept of axSpA, which indicates axSpA patients without definite sacroiliitis on conventional radiographs. Totally 50 individuals with chronic low back pain were not diagnosed with axSpA, and disc degeneration, muscle strain, or other factors may be etiological for their clinical symptoms.

### Clinical and WBMRI examinations

Clinicodemographic data were collected, including gender, age, smoking history, duration of symptoms, C-reactive protein (CRP) levels, erythrocyte sedimentation rate (ESR), HLA-B27, current medication, and concomitant symptoms. The Ankylosing Spondylitis Disease Activity Score (ASDAS) (12) was used to assess the severity of spondyloarthritis. Clinical enthesitis was evaluated using a modified Maastricht Ankylosing Spondylitis Enthesitis Score (MASES) and Leeds Enthesitis Index (LEI). The clinical enthesitis score is the sum of MASES and LEI scores. All patients underwent WBMRI within a week.

WBMRI was performed on a Philips 3T Ingenia unit (Philips Healthcare, Best, Netherlands) using phased-array coils with patients in the supine position. A coronal T1-weighted mDixon sequence (FOV, 777 × 1,674 mm; matrix size, 408 × 408; thickness, 5 mm; TR/TE, 4/2 ms; slice gap, 0.5 mm) and short τ inversion recovery (STIR) sequence (FOV, 784 × 1m674 mm; matrix size, 408 × 359; thickness, 5 mm; TR/TE, 10,877/70 ms; slice gap, 0.5 mm) were performed. The total scan time was 25 min and was well tolerated by the study participants.

### Whole-body MRI assessment

**Figure 1:**
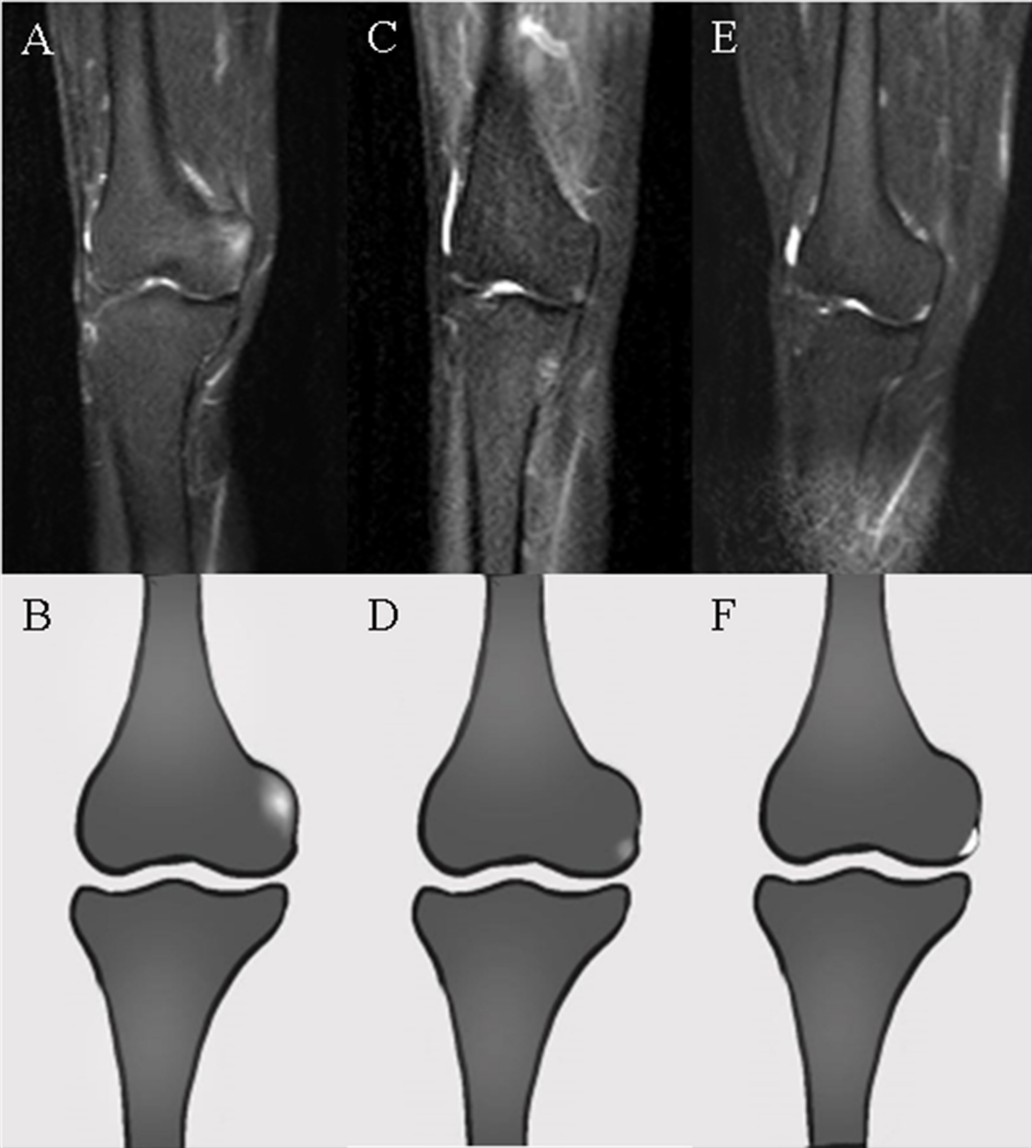
WBMRI images and ideogram of the medial femoral condyle. **(A, B)** Hyperintensity with ill-defined margins at the adductor tubercle of the medial femoral condyle on STIR images representing osteitis in enthesitis. **(C, D)** Slightly hyperintense signals with ill-defined margins at the outer inferior border of the medial femoral condyle, considered inflammation of the medial joint capsule (deep layer of the medial collateral ligament) or medial meniscal attachment. **(E, F)** Clear hyperintensity adjacent to the border of the medial femoral condyle, representing joint fluid rather than enthesitis.

Radiologists evaluated the acquired WBMRI images based on preliminary standards for WBMRI in inflammatory arthritis that were developed with further iteration by the Working Group meetings at the Outcome Measures in Rheumatology (OMERACT): MRI-WIPE (13, 14) and the WBMRI index for inflammation of peripheral joints and enthesis definitions, scoring methodology, and image examples established by OMERACT. Enthesitis was diagnosed based on high signal intensity on short TI inversion recovery (STIR) images obtained with a corresponding signal loss on T1-weighted images within the bone marrow (bone marrow edema [BME]) or the surrounding soft tissue (soft tissue edema) (15). Osteitis should be ill-defined in the bone marrow. Hyperintense lesions of the bone with clear borders need to be considered bone cystic changes or joint effusion rather than active inflammation. Osteitis should be assessed in the bone from the entheseal insertion to a depth of 1 cm on all available images. Soft tissue inflammation was assessed inside the dense fibrous connective tissue of the enthesis as well as in its immediate surroundings to a distance of 1 cm from the entheseal insertion. **Figure 1** depicts enthesitis of the medial femoral condyle.

The specific rules of severity score for bone marrow edema and surrounding soft tissue were as follows:

a. Bone marrow edema: 0, no edema; 1, 1%–50% of the bone edematous; 2, 51%–100% of the bone edematous.
b. Soft tissue inflammation: 0, normal; 1, mild or moderate; 2, severe.
c. Sum score (single enthesitis) = a+b
d. In case a reader hesitated whether to score a given lesion at 1 or 0, a score of 0 was considered. For a lesion that was borderline between 1 and 2, lesion intensity may be considered.

**Figure 2:**
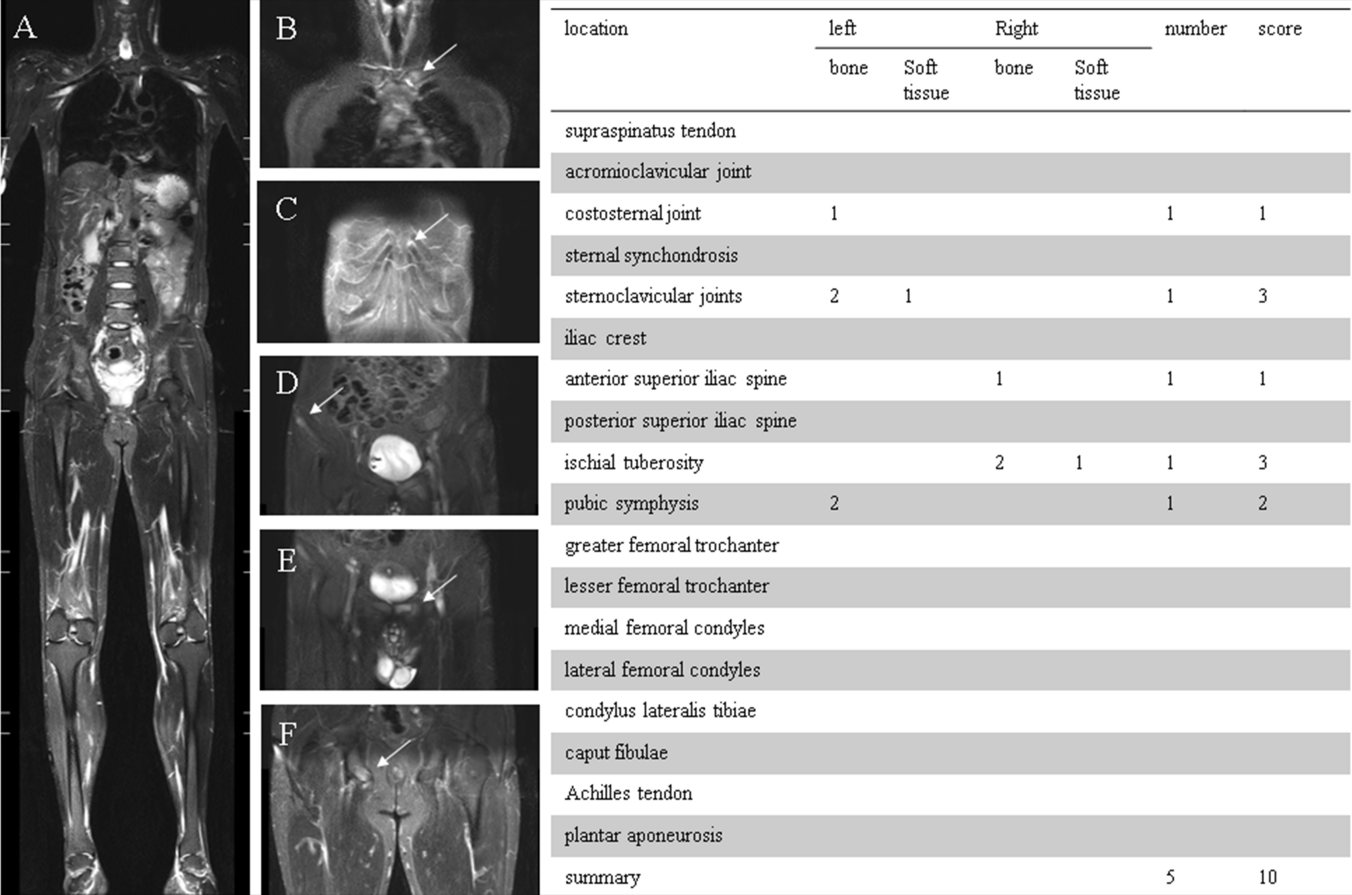
**(A)** 36-year-old man, HLA-B27 positive, was classified as ankylosing spondylitis (AS). **(A)** Coronal STIR whole-body MRI image demonstrated entheseal sites from shoulder to heel. **(B)** Enthesitis of the left sternoclavicular joint (including bone marrow edema and joint effusion). **(C)** Enthesitis of the left costosternal joint. **(D)** Enthesitis of the right anterior superior iliac spine (bone marrow edema). **(E)** Enthesitis of the pubic symphysis (bone marrow edema). **(F)** Enthesitis of the right ischial tuberosity (including bone marrow edema and soft tissue inflammation). The table on the right indicates the numbers and scores of entheses.

The following 18 locations were evaluated and subdivided into five regions (**Figure 2**): region 1 (shoulder), acromioclavicular joint and supraspinatus tendon insertion at the humerus; region 2 (anterior chest wall), costosternal, manubriosternal, and sternoclavicular joints; region 3 (pelvis), iliac crest, anterior superior iliac spine, posterior superior iliac spine, ischial tuberosity, pubic symphysis, greater femoral trochanter, and lesser femoral trochanter; region 4 (knee), medial femoral condyles, lateral femoral condyles, condyles lateralis tibiae, and caput fibulae; region 5 (foot), insertion of the Achilles tendon and plantar aponeurosis.

The elbow joint, hands, and toes were not imaged because of coil limitations. Forty-seven sites were assessed in each patient, with scores of 0–188. All MR images were scored independently by two musculoskeletal radiologists, who were blinded to clinical and biochemical information. The obtained images were evaluated in random order. Reading sessions were performed with a PACS (Picture Archiving and Communication Systems) workstation using two high-resolution monitors. After read completion, cases with discrepant results were sent for discussion to both readers, who had to select a consensual score.

### Statistical analysis

SPSS version 25 (SPSS, IL, USA) was used to perform all statistical analyses. The Kolmogorov–Smirnov test was used to assess data normality. Data were presented as mean ± standard deviation (SD), median and interquartile range, or percentage (%). Patient characteristics among the AS, nr-axSpA, and control groups were compared by the independent-sample t-test and the Wilcoxon rank-sum test. P < 0.05 indicated statistical significance. Associations of the number and score of peripheral enthesitis with clinical enthesitis, ASDAS, CRP, ESR, and disease duration were assessed by Spearman’s correlation analysis. Receiver operating characteristic (ROC) curve analysis, with area under the curve (AUC) and 95% confidential interval (CI) determinations, was performed to evaluate the role of peripheral enthesitis in the diagnosis of axSpA. AUROCs were compared by the two-sided DeLong test. Reliability analyses included intrareader intraclass correlation coefficients (ICC) (two-way mixed model, absolute agreement definition). Missing values were imputed by multiple imputation. The sample size was estimated based on the best compromise between the sensitivity and specificity of WBMRI enthesitis. Based on the results of preliminary experiments as well as previous studies (16, 17), a sensitivity of 0.75 and a specificity of 0.9 required at least 45 axSpA patients.

## Results

### Patient characteristics

Patients with axSpA were 33.2 ± 8.4 years old, including 42 men (70%) and 18 women (30%). The median disease duration was 5.0 years (interquartile range of 7). ASDAS scores were 2.31 ± 1.0. Twenty-six patients (43.3%) fulfilled the ankylosing spondylitis (AS) criteria; 34 patients (56.7%) fulfilled the ASAS classification criteria but had no bone destruction of the sacroiliac joint surface diagnosed with nr-axSpA. Thirty-two patients had biological agent treatment (including TNF inhibitors and IL-17-blocking monoclonal antibodies) within a few months after the diagnosis. Totally 50 controls without axSpA had a mean age of 34.7 years. Their median disease duration was 3.0 years (interquartile range 6.0). The basic data of the participants are shown in the **Table 1**.

**Table 1:** Baseline characteristics in axSpA patients and control controls.

Characteristics	axSpA (n = 60)	AS (n = 26)	nr-axSpA (n = 34)	Control group (n = 50)	P^*^
Male, n (%)	42 (70)	20 (76.9)	22 (64.7)	22 (44%)	0.006
Age, years, mean (SD)	33.2 (8.4)	35.6 (8.1)	30.8 (7.0)^**^	34.7 (6.4)	0.302
Weight, kg, mean (SD)	74.5 (15.3)	73.5 (10.6)	75.3 (18.0)	60.2 (24.1)	0.011
Disease duration, year, median (IQR)	5.0 (7.0)	5 (9.7)	3 (4.0)^**^	3.0 (6.0)	0.317
HLA-B27–positive, n (%)	49 (81.7)	23 (88.4)	26 (76.4)	4 (8)	<0.001
ASDAS, mean (SD)	2.3 (1.0)	2.4 (1.0)	2.1 (0.9)	0.7 (0.7)	<0.001
CRP, mg/L, mean (SD)	5.9 (9.4)	6.9 (8.4)	5.2 (10.2)	2.1 (3.2)	0.025
ESR, mg/L, mean (SD)	19.5 (17.3)	17.9 (14.8)	20.4 (20.1)	5 (1.1)	<0.001

axSpA, axial spondyloarthritis; AS, ankylosing spondylitis; nr-axSpA, non-radiographic axial spondyloarthritis; ASDAS, Ankylosing Spondylitis Disease Activity Score; CRP, C-reactive protein; ESR, erythrocyte sedimentation rate; SD, standard deviation; IQR, interquartile range.

*Comparison between the axSpA and control group.

**Comparison between AS and nr-axSpA, P < 0.05.

### Distribution of WBMRI enthesitis in the axSpA and control groups

**Figure 3:**
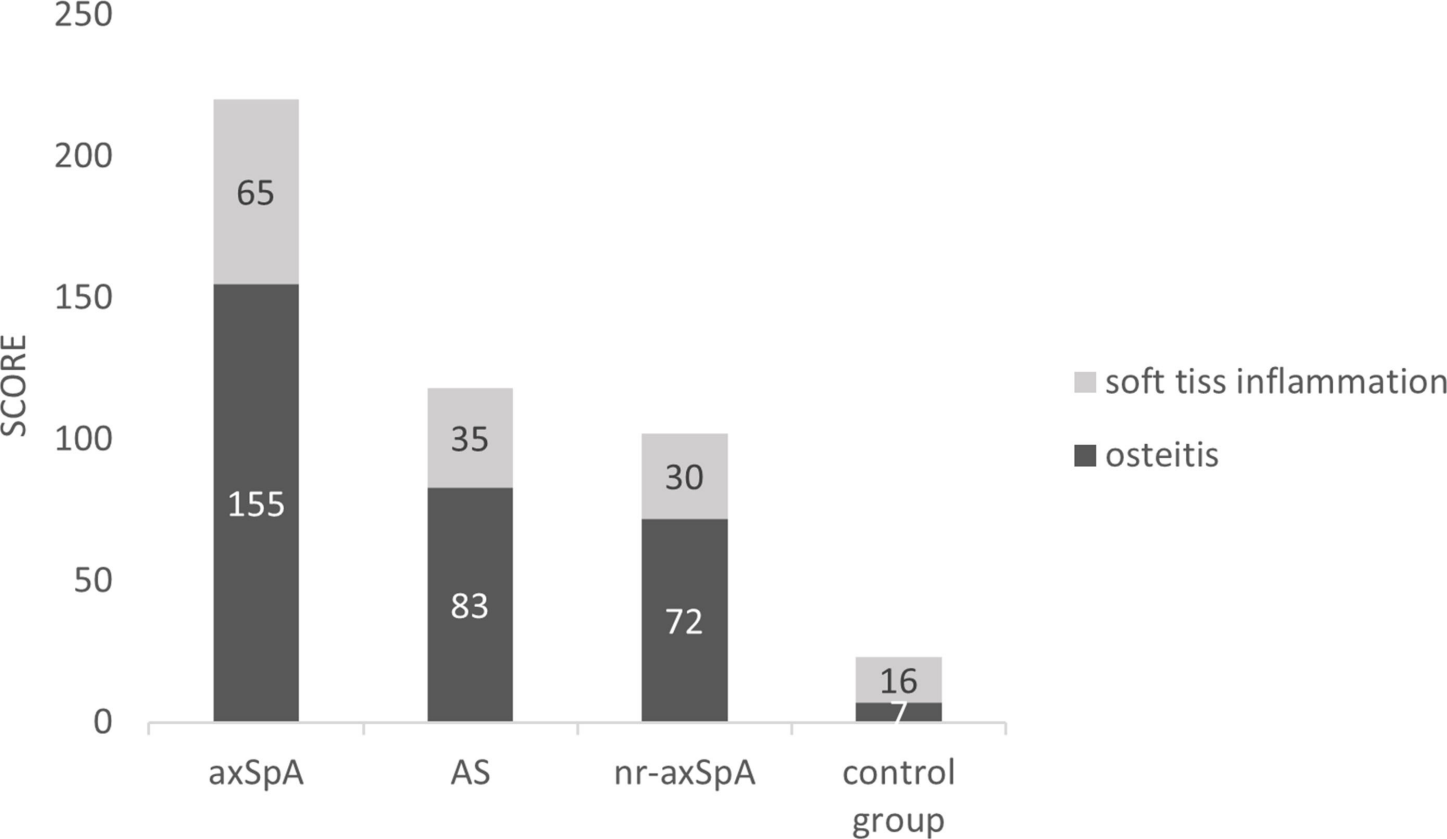
Soft tissue inflammation and osteitis scores in the axSpA, AS, nr-axSpA, and control groups. axSpA, axial spondyloarthritis. AS, ankylosing spondylitis. nr-axSpA, non-radiographic axial spondyloarthritis.

A total of 2,820 (47 × 60) entheseal sites were evaluated in axSpA patients, and WBMRI identified 155 enthesitis sites. The AS and nr-axSpA groups showed similar numbers of enthesitis (80 vs. 75). The pelvis had the maximum number of enthesitis (52, 33.5%) in axSpA patients. The pelvis was also the region with the most enthesitis sites in patients with AS and nr-axSpA (29 vs. 23). The foot had the least number of enthesitis (14, 9.0%) lesions in axSpA patients, whereas the Achilles tendon had two enthesitis sites. The total enthesitis scores of axSpA patients were 220 (mean 3.7 ± 3.1). The most frequently scored entheseal sites were located in the pelvis (78, 35.4%). WBMRI identified 23 enthesitis sites in the control group. The score of each enthesitis site in the control group was 1. The shoulder and pelvis had the maximum number of enthesis sites (8, 33%) in the control group. No enthesitis was detected in the anterior chest wall. The differences in subtypes based on entheseal score were significant between axSpA patients and the control group. The osteitis score accounted for a large proportion of total score in axSpA patients (155/220, 70.5%). However, the osteitis score accounted for a smaller proportion of total score in the control group (7/23, 30.4%). The complete numbers and scores of enthesitis are shown in **Table 2** and **Figure 3**. The number of WBMRI enthesitis correlated with clinical enthesitis (r = 058; P = 0.001).

**Table 2:** Numbers and scores of enthesitis in the axSpA, AS, nr-axSpA, and control groups.

	Entheseal sites	axSpA (60)	AS (26)	Nr-axSpA (34)	Controls (50)
**Shoulder**	Acromioclavicular joint	14 (19)	7 (9)	7 (10)	4 (4)
	Supraspinatus tendon	12 (16)	7 (10)	5 (6)	4 (4)
		**26 (35)**	**14 (19)**	**12 (16)**	**8 (8)**
**Anterior chest wall**	Costosternal joint	15 (20)	10 (15)	5 (5)	0 (0)
	Manubriosternal joint	9 (11)	4 (4)	5 (7)	0 (0)
	Sternoclavicular joints	17 (31)	8 (15)	9 (16)	0 (0)
		**41 (62)**	**22 (34)**	**19 (28)**	**0 (0)**
**Pelvis**	Iliac crest	1 (3)	1 (3)	0 (0)	0 (0)
	Anterior superior iliac spine	4 (4)	1 (1)	3 (3)	2 (2)
	Posterior superior iliac spine	4 (5)	2 (2)	2 (3)	0 (0)
	Ischial tuberosity	13 (22)	6 (11)	7 (11)	1 (1)
	Pubic symphysis	10 (17)	8 (15)	2 (2)	0 (0)
	Greater femoral trochanter	19 (25)	11 (13)	8 (12)	5 (5)
	Lesser femoral trochanter	1 (2)	0 (0)	1 (2)	0 (0)
		**52 (78)**	**29 (45)**	**23 (33)**	**8 (8)**
**Knee**	Medial femoral condyles	16 (17)	5 (5)	11 (12)	3 (5)
	Lateral femoral condyles	2 (3)	0 (0)	2 (3)	0 (0)
	Condylus lateralis tibiae	3 (3)	3 (3)	0 (0)	2 (2)
	Caput fibulae	1 (1)	1 (1)	0 (0)	0 (0)
		**22 (24)**	**9 (9)**	**13 (15)**	**5 (5)**
**Foot**	Achilles tendon	2 (4)	2 (4)	0 (0)	0 (0)
	Plantar aponeurosis	12 (17)	4 (7)	8 (10)	2 (2)
		**14 (21)**	**6 (11)**	**8 (10)**	**2 (2)**
**Summary**		**155 (220)**	**80 (118)**	**75 (102)**	**23 (23)**
**Mean**		2.5 (3.7)^**^	3.1 (4.5)^*^	2.2 (3.0)^*^	0.4 (0.4)^**^

*Comparison of numbers and scores between the AS and nr-axSpA groups, P = 0.09 (0.05).

**Comparison of numbers and scores between axSpA and LBP patients, P < 0.001 (0.001).

axSpA, axial spondyloarthritis; AS, ankylosing spondylitis; nr-axSpA, non-radiographic axial spondyloarthritis.

Data are number (score).

The bold values represent the enthesitis at each assessed location (shoulder, anterior chest wall, pelvis, knee and foot).

WBMRI identified enthesitis in 78.3% of axSpA patients (n = 47). The pelvic region was the most frequently affected body region, with 51.6% involved patients (n = 31). The proportion of axSpA patients with enthesitis in the foot region was the least (18.3%, n = 11). WBMRI identified enthesitis in 32% of patients without axSpA (n = 16). Detailed results are presented in **Table 3**.

**Table 3:** Numbers and proportions of patients with enthesitis in the axSpA and control groups.

Location	axSpA	AS	nr-axSpA	Controls	P^*^
Shoulder	21 (35.0%)	11 (42.3%)	10 (29.4%)	8 (16%)	0.015
Anterior chest wall	23 (38.3%)	11 (42.3%)	12 (35.3%)	0 (0%)	<0.001
Pelvis	31 (51.6%)	14 (53.8%)	17 (50.0%)	5 (10%)	<0.001
Knee	14 (23.3%)	6 (23.0%)	8 (23.5%)	4 (8%)	0.030
Foot	11 (18.3%)	6 (23.1%)	5 (14.7%)	1 (2%)	0.002
Total	47 (78.3%)	21 (80.7%)	26 (76.4%)	16 (32%)	<0.001

axSpA, axial spondyloarthritis; AS, ankylosing spondylitis; Nr-axSpA, non-radiographic axial spondyloarthritis.

*Comparison between the axSpA and control groups.

### Diagnostic value of enthesitis for axSpA diagnosis

The sensitivities, specificities, positive and negative likelihood ratios, and predictive values of each peripheral area are presented in **Table 4**. Enthesitis of the pelvis showed the highest sensitivity (51.67%), and specificity, LR+, LR−, PPV, and NPV were 90%, 5.17, 0.53, 86.11%, and 60.81%, respectively. Foot enthesitis had the lowest sensitivity (18.33%) in the diagnosis of axSpA, with a specificity of 98%. Anterior chest wall enthesitis had the highest specificity (100%), as no anterior chest wall inflammation was present in controls. The specificity was lowest for the shoulder (84%).

**Table 4:** Sensitivity (SN), specificity (SP), and positive and negative likelihood ratios (LRs) and positive and negative predictive values (PVs) of enthesitis in different areas for the diagnosis of axSpA.

Location	SN (%)	SP (%)	LR+	LR-	PPV (%)	NPV (%)
Shoulder	35.00	84	2.18	0.72	73.33	51.80
Anterior chest wall	38.33	100		0.61	100	57.47
Pelvis	51.67	90	5.17	0.53	86.11	60.81
Knee	23.33	92	2.91	0.83	77.78	50.00
Foot	18.33	98	9.19	0.83	91.67	50.00
Total	78.33	68	2.44	0.32	74.60	72.34

**Figure 4:**
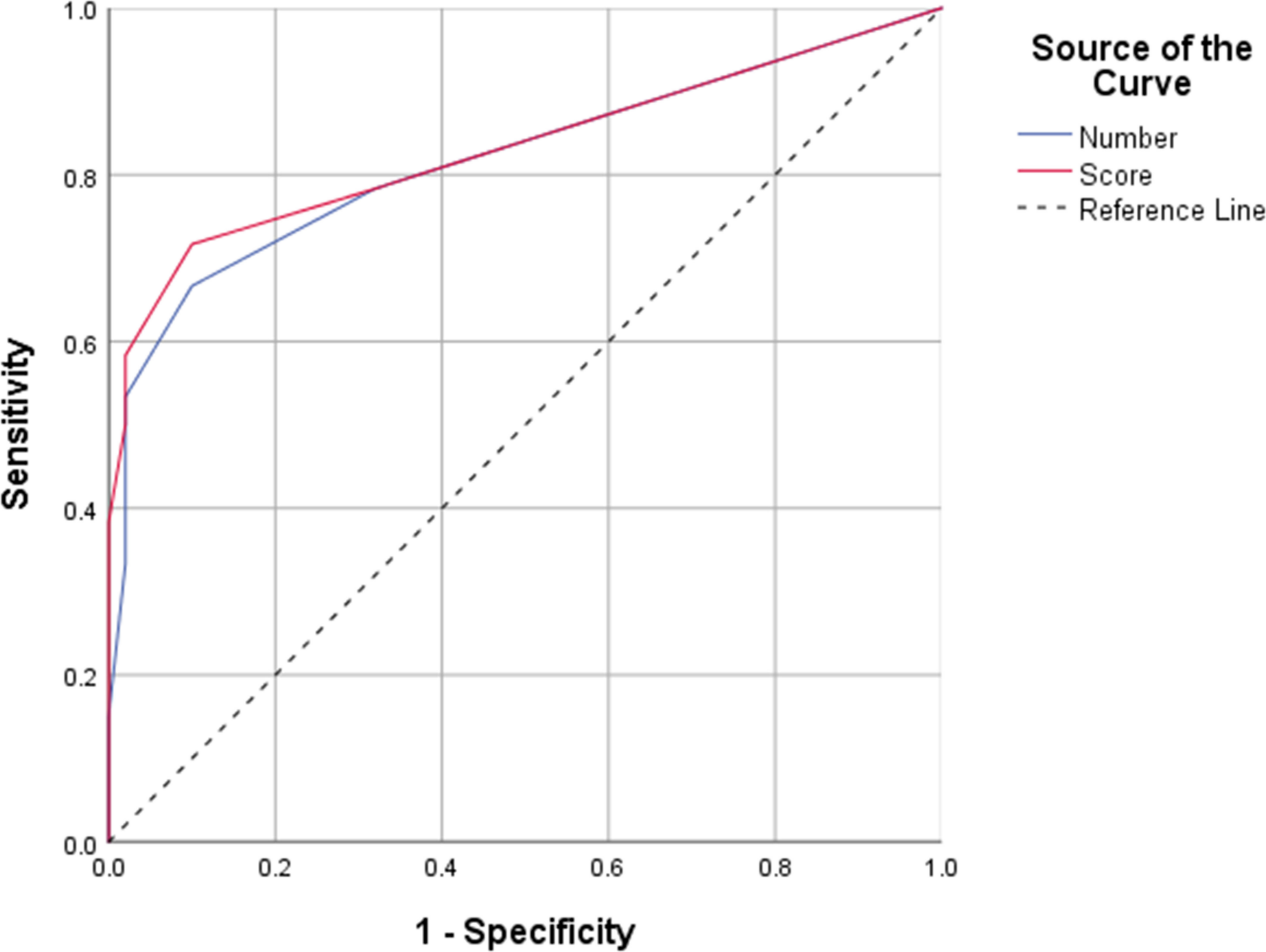
Receiver operating characteristic (ROC) curve analysis of the number and score of whole-body enthesitis in the diagnosis of axial spondyloarthritis.

ROC curves were generated to determine the efficiency of peripheral enthesitis number and score in axSpA diagnosis. The AUC for the number of enthesitis was 0.819 (95% CI 0.739–0.899), and that for the enthesitis score was 0.832 (95% CI 0.754–0.910), indicating statistically significant differences (P = 0.025). Based on the Youden index and clinical need, an enthesitis number or score of 3 as cutoff may have the greatest value for axSpA diagnosis (number of enthesitis: sensitivity, specificity, LR+, LR−, and Youden index were 53.33%, 98%, 26.6, 0.47, and 0.51, respectively; score of enthesitis: sensitivity, specificity, LR+, LR−, and Youden index were 58.33%, 98%, 29.1, 0.42, and 0.56, respectively). The ROC for peripheral enthesitis is shown in **Figure 4**. The number/score of peripheral enthesitis sites was not correlated with Ankylosing Spondylitis Disease Activity Score (ASDAS) (r = 0.16, P = 0.22 and r = 0.15, P = 0.22, respectively), CRP (r = 0.12, P = 0.33 and r = 0.15, P = 0.24, respectively), ESR (r = 0.25, P = 0.10 and r = 0.30, P = 0.06, respectively), and disease duration (r = 0.11, P = 0.38 and r = 0.08, P = 0.50, respectively).

### Readability of enthesis detection on WBMRI scans

Intrareader reproducibility for WBMRI assessment was analyzed, and an intrareader ICC for the number of enthesitis sites on WBMRI scans was 0.82 (0.68–0.90). The inter-reader reliability for enthesitis score was 0.88 (0.76–0.94). The ICCs for separate entheseal sites are shown in **Supplementary Table 1**.

## Discussion

Taking the comprehensive diagnosis of clinicians as the gold standard, we concluded that peripheral enthesitis detected by whole-body magnetic resonance imaging (WBMRI) could well distinguish axial spondyloarthritis (axSpA) patients from individuals with non-specific chronic low back pain. The score of peripheral enthesitis had slightly higher diagnostic efficacy than the latter number. There were differences in the distributions and scores of enthesitis between the axSpA and control groups. No associations were found of enthesitis number and score with clinical characteristics (CRP, ESR, ASDAS, and disease duration). We speculate that peripheral enthesitis is relatively independent in patients with axSpA and cannot be well reflected by the activity index. Poggenborg et al. also demonstrated no significant correlations between peripheral WBMRI score and patient characteristics or clinical parameters of disease activity (18).

In this study, the distribution of peripheral enthesitis in patients with axSpA was described. The order of body parts affected by enthesitis from most to least was pelvis, anterior chest wall, shoulder, knee, and foot. A literature reported that the pelvic region is the most frequently affected body region in terms of enthesitis (19). Another research showed that enthesitis most frequently occurs at the greater femoral trochanter, supraspinatus, and Achilles tendon insertions (20). Inflammatory involvement of the manubriosternal joint by various imaging modalities has been reported in 39% to 85% of AS patients (21). We found that foot lesions were not as much as expected, although the heel is the most frequent enthesitis location in previous reports (8). The reasons for discrepant conclusions could be broad inclusion criteria, longer symptom duration, and differences in medication use. However, this reflects clinical practice. Another important reason may be the different distributions of enthesitis in axSpA patients and peripheral spondyloarthritis (PSpA) patients as subjects in other studies, which may also affect subsequent treatment decisions in the case of diverse biological agents to choose from.

Although enthesitis is the core pathological change of SpA, its diagnostic value in different parts may be different. The specificity of peripheral enthesitis areas ranked from highest to lowest as anterior chest wall (100%), foot (98%), knee (92%), pelvis (90%), and shoulder (84%) in this study. That is, when the diagnosis is equivocal, detecting anterior chest wall inflammation can greatly improve diagnostic confidence. The anterior chest wall is rarely damaged by repeated mechanical stress in healthy individuals, which may be one of the reasons for its high specificity. The shoulder had the lowest specificity, because the supraspinatus tendon humeral insertion and the acromioclavicular joint are most affected by degeneration and sports injuries, implying that injuries are most easily observed in normal individuals. Enthesitis (or enthesopathy) refers to all pathological abnormalities of insertions, including inflammatory changes and degenerative events (22). It was detected on the acromioclavicular joint, supraspinatus tendon, anterior superior iliac spine, ischial tuberosity, greater femoral trochanter, medial femoral condyles, condylus lateralis tibiae, and plantar aponeurosis in the control group by WBMRI. Greater trochanteric pain syndrome (GTPS) (23), plantar fasciitis, and proximal iliotibial band syndrome might explain the abnormal signal in the greater femoral trochanter, plantar aponeurosis, and anterior superior iliac spine. Degeneration and inappropriate or excessive exercise may also be an important cause of enthesopathy in the control group. Therefore, when interpreting enthesitis, it is essential to combine the patient’s motor history to make a comprehensive diagnosis.

Studies have explored the diagnostic efficacy of peripheral enthesitis. Jans et al. concluded that pelvic enthesitis on MRI scans of the sacroiliac joint has a high specificity for the diagnosis of SpA, and the concomitant presence of more than one enthesitis site further increases this specificity, while specificity was only 24.4% (16). De Miguel et al. showed that ultrasound-based enthesis score could be a valid tool for the diagnosis of SpA, with 83.3% sensitivity and 82.8% specificity, even in the absence of clinical findings (24). Power Doppler ultrasonography (PDUS) of entheses (eight sites) in individuals with inflammatory back pain suggestive of axSpA in the DESIR cohort showed that although enthesitis prevalence was low (14.4%), its specificity for categorizing patients as having axSpA based on ASAS criteria was high (83.5%). A positive predictive value for meeting the ASAS criteria for axSpA was 69%. PDUS of entheses may therefore facilitate early diagnosis in patients not fulfilling the ASAS classification criteria (17).

The hypothesis of this study was that enthesitis caused by non-immune reasons may occur in non-SpA patients, but multiple enthesitis at different sites rarely occurs in cases without concurrent SpA. Therefore, it is essential to determine how much enthesitis can maximally assist in SpA diagnosis. We finally determined that three enthesitis sites and enthesitis score may have the greatest value for axSpA diagnosis, with acceptable sensitivity (53.3%–58.3%) and high specificity (98%). Using two enthesitis sites (number/score) as cutoff would increase sensitivity to 66.67%/71.67%, but specificity would decrease to 90%/90%. The specificity would decrease to 68% if one enthesitis site is used as cutoff. Overall, this study revealed peripheral enthesitis as a low-sensitivity and high-specificity tool for the diagnosis of axSpA. In daily clinical practice, clinicians should pay more attention to the specificity of peripheral enthesitis, that is, whether peripheral enthesitis could provide confidence in enthesitis diagnosis.

Scoring of enthesitis may be more helpful in axSpA diagnosis from non-SpA individuals with low back pain. This work showed that the enthesitis score had better diagnostic efficiency than the number of enthesitis sites. When a specific cutoff is chosen, some missed patients may be reclassified as axSpA cases because of the score, while the enthesitis score in the control group is low, which has little impact on specificity. In addition, the subtypes of enthesitis based on the disease score differed between enthesitis patients and controls, which is also helpful in differential diagnosis. Enthesitis detected in the control group more frequently had soft tissue inflammation (16, 69.5%) than that of axSpA patients (65, 29.5%). This finding indicated that osteitis is more pronounced in SpA patients. This potential in differentiating inflammatory etiology from degenerative and other causes of enthesitis has initially appeared but needs to be appraised in a larger number of patients (25).

WBMRI is broadly utilized, including in oncology, pediatrics, and rheumatological and musculoskeletal conditions. The readability of MRI scans varies substantially. In a study by Wetterslev et al., pelvic and hip joint inflammation was evaluated with two scoring systems (MRI-WIPE and HIMRISS) on whole-body MRI images. The methods showed mostly good agreement, which varied from poor to very good between readers (26). Weckbach et al. (27) reported good quality at centrally located joints and lower quality at distal peripheral joints. The intrareader ICC for the total number of entheses with enthesitis on WBMRI scans (WBMRI score) was 0.82 in this study. Thick slices and only using coronal scans were the main reasons for inconsistency among readers, but this should not greatly affect enthesitis identification.

Overall, the feasibility of WBMRI as a clinical tool for SpA needs further evaluation and the following two points need to be discussed: 1) since WBMRI examination is expensive and time-consuming, is it worthy that patients pay these costs to obtain more comprehensive information of peripheral enthesitis? 2) The readability of WBMRI varied substantially in different studies, which in detecting enthesitis at different sites also varied. Therefore, the readability of MRI images (especially the joints located at the edges of the images) needs to be further improved.

While this study was well planned prospectively, it had some limitations. This was a real-world study, with diverse disease courses and treatments for various patients. Further research may be needed to explore the distribution and outcome characteristics of enthesitis under different disease courses and treatments. No further clinical diagnosis of enthesitis in the control group was made in this study. The scanning strategy needs to be further improved, seeking to improve the image quality without increasing the scanning time too much. Besides, evidence suggests that contrast-agent use improves the sensitivity and specificity of MRI for routine assessment of individual patients (28). Limited by selection bias because WBMRI is not a routine examination, the prevalence of spondyloarthritis in LBP patients was higher in this study compared with other reports, which may affect the reliability of positive and negative predictive values. Finally, follow-up observation of larger samples is needed to verify the present conclusions.

## Conclusion

In conclusion, the distribution of peripheral enthesitis can be adequately assessed by whole-body MRI, and peripheral enthesitis can facilitate the diagnosis of spondyloarthritis. Entheses at various locations have different values for the diagnosis of axial spondyloarthritis. Enthesitis scoring may provide a more accurate assessment and diagnosis for axSpA than enthesitis site counting. Finally, although enthesitis has high specificity, it is still necessary to explain the abnormal signal of entheseal sites in combination with clinical history to avoid overdiagnosis.

## Data availability statement

The raw data supporting the conclusions of this article will be made available by the authors, without undue reservation.

## Ethics statement

The studies involving human participants were reviewed and approved by the ethics committee of the Tianjin First Central Hospital (No.2021N156KY). The patients/participants provided their written informed consent to participate in this study. Written informed consent was obtained from the individual (s) for the publication of any potentially identifiable images or data included in this article.

## Author contributions

ZG contributed to this work in planning, analyzing, evaluating, and writing the paper. BL participated in experimental design and machine operation. Other authors contributed to the manuscript review and image analysis. All authors agree to be accountable for the content of the work. All authors contributed to the article and approved the submitted version.

## Funding

This study was funded by funded by Tianjin Key Medical Discipline(Specialty) Construction Project (TJYXZDXK-041A), and Tianjin health bureau scientific foundation of China (ZC20043).

## Acknowledgments

We would like to acknowledgement all colleagues in the radiology department, especially those in the MRI department, for their contribution to the scanning.

## Conflict of interest

Author ZS was employed by Philips Healthcare.

The remaining authors declare that the research was conducted in the absence of any commercial or financial relationships that could be construed as a potential conflict of interest.

## Publisher’s note

All claims expressed in this article are solely those of the authors and do not necessarily represent those of their affiliated organizations, or those of the publisher, the editors and the reviewers. Any product that may be evaluated in this article, or claim that may be made by its manufacturer, is not guaranteed or endorsed by the publisher.

## Abbreviations

axSpA, axial spondyloarthritis; WBMRI, whole-body MRI; LBP, low back pain; AS, ankylosing spondylitis; Nr-axSpA, non-radiographic axial spondyloarthritis; ROC, receiver operating characteristic; AUC, area under curve; ASAS, Assessment of Spondyloarthritis International Society; ESR, erythrocyte sedimentation rate; CRP, C-reactive protein; ASDAS, Ankylosing Spondylitis Disease Activity Score; STIR, short τ inversion recovery; OMERACT, Outcome Measures in Rheumatology Clinical Trials; BME, bone marrow edema; MRI-WIPE, Whole-Body MRI Score for Inflammation in Peripheral Joints and Entheses in Inflammatory Arthritis; HIMRISS, hip inflammation MRI scoring system.
