# Supplementary material for: Peripheral enthesitis assessed by whole-body MRI in axial spondyloarthritis: Distribution and diagnostic value
Source: Front Immunol. 2022 Aug 23;13:976800. doi: 10.3389/fimmu.2022.976800 (PMC9446460; doi:10.3389/fimmu.2022.976800)
Supplement: Supplementary file 1 [file Table_1.docx]

Supplementary Material

# Supplementary table 1 intraclass correlation coefficient (ICC) of separate entheseal site. two-way mixed effect model (absolute agreement) single measure ICC.

|  | Entheseal sites | ICC-score | CI |
| --- | --- | --- | --- |
| **shoulder** | acromioclavicular joint | 0.91 | 0.68-0.97 |
|  | Supraspinatus tendon | 0.57 | 0.37-0.72 |
| **Anterior chest wall** | Costosternal joint | 0.89 | 0.69-0.93 |
|  | Manubriosternal joint | 0.88 | 0.80-0.92 |
|  | sternoclavicular joints | 0.82 | 0.72-0.89 |
| **pelvis** | iliac crest | 0.82 | 0.72-0.89 |
|  | Anterior superior iliac spine | 0.49 | 0.16-0.70 |
|  | posterior superior iliac spine | 0.80 | 0.59-0.88 |
|  | ischial tuberosity | 0.69 | 0.53-0.80 |
|  | pubic symphysis | 0.94 | 0.90-0.96 |
|  | greater femoral trochanter | 0.85 | 0.76-0.90 |
|  | lesser femoral trochanter | 1.0 |  |
| **knee** | medial femoral condyles | 0.76 | 0.63-0.85 |
|  | lateral femoral condyles | 1.0 |  |
|  | condylus lateralis tibiae | 0.71 | 0.56-0.81 |
|  | caput fibulae | 0.66 | 0.48-0.78 |
| **foot** | Achilles tendon | 0.78 | 0.64-0.87 |
|  | plantar aponeurosis | 0.90 | 0.83-0.94 |
